# MicroRNAs and cytokines as potential predictive biomarkers for COVID-19 disease progression

**DOI:** 10.1038/s41598-023-30474-6

**Published:** 2023-03-02

**Authors:** Hatem A. Mohamed, Aya Eid Abdelkafy, Rasha M. M. Khairy, Salama R. Abdelraheim, Bothina Ahmed Kamel, Heba Marey

**Affiliations:** 1 Department of Biochemistry, Faculty of Medicine, Minia University, Minia, Egypt; 2 Department of Microbiology and Immunology, Faculty of Medicine, Minia University, Minia, 61511 Egypt

**Keywords:** Immunology, Microbiology, Biomarkers, Medical research, Pathogenesis

## Abstract

Host microRNAs can influence the cytokine storm associated SARS-CoV-2 infection and proposed as biomarkers for COVID-19 disease. In the present study, serum MiRNA-106a and miRNA-20a were quantified by real time-PCR in 50 COVID-19 patients hospitalized at Minia university hospital and 30 healthy volunteers. Profiles of serum inflammatory cytokines (TNF-α, IFN-γ, and IL-10) and TLR4 were analyzed by Eliza in patients and controls. A highly significant decrease (*P* value = 0.0001) in the expressions of miRNA-106a and miRNA-20a was reported in COVID-19 patients compared to controls. A significant decrease in the levels of miRNA-20a was also reported in patients with lymphopenia, patients having chest CT severity score (CSS) > 19 and in patients having O_2_ saturation less than 90%. Significantly higher levels of TNF-α, IFN-γ, IL-10 and TLR4 were reported in patients compared to controls. IL-10 and TLR4 levels were significantly higher in patients having lymphopenia. TLR-4 level was higher in patients with CSS > 19 and in patients with hypoxia. Using univariate logistic regression analysis, miRNA-106a, miRNA-20a, TNF-α, IFN-γ, IL-10 and TLR4 were identified as good predictors of disease. Receiver operating curve showed that the downregulation of miRNA-20a in patients having lymphopenia, patients with CSS > 19 and patients with hypoxia could be a potential biomarker with AUC = 0.68 ± 0.08, AUC = 0.73 ± 0.07 and AUC = 0.68 ± 0.07 respectively. Also, ROC curve showed accurate association between the increase of serum IL-10 and TLR-4 and lymphopenia among COVID-19 patients with AUC = 0.66 ± 0.08 and AUC = 0.73 ± 0.07 respectively. ROC curve showed also that serum TLR-4 could be a potential marker for high CSS with AUC = 0.78 ± 0.06. A negative correlation was detected between miRNA-20a with TLR-4 (r = − 0.30, *P* value = 0.03). We concluded that, miR-20a, is a potential biomarker of COVID-19 severity and blockade of IL-10 and TLR4 may constitute a novel therapy for COVID-19 patients.

## Introduction

In December 2019, unexplained cases of pneumonia have appeared in Wuhan City, China. Chinese scientists identified this infection as a novel coronavirus infection, labeled as severe acute respiratory syndrome coronavirus 2 (SARS-CoV-2)^1^. Its name was then changed to coronavirus disease 2019 (COVID-19) by the world health organization in February 2020^2^. Infection is transmitted by large droplets generated during coughing or sneezing by symptomatic patients and asymptomatic people^3^. Once the virus gains access into the target cell, the host immune cells recognize the virus antigens eliciting the immune response. However many studies suggest that patients with SARS-CoV or MERS-CoV have dysregulated immune response and cytokine secretion, where secretion of pro-inflammatory cytokines like TNF-α, IL-6, IL-1β and IFN-α/-δ, with reduced anti-inflammatory cytokines occur^4^. These high levels of cytokines were associated with multi-organ dysfunctional syndrome (MODS) and acute respiratory distress syndrome (ARDS)^5^. Similarly, in COVID-19 patients, release of cytokines is high leading to ARDS, which is fatal to critically ill patients^6,7^. This exaggerated immune reaction called cytokine release syndrome (CRS)^8^. TLR4 is considered as an important player in the study of COVID-19 infection, which induces the release of pro-inflammatory mediators to overcome infection^9^. There is accumulating data indicate that, SARS-CoV-2 can bind to TLR4 and treatment with resatorvid (a TLR4 specific inhibitor) has been reported to inhibit the release of IL-1β^10^. Although most TLRs are transmembrane receptors, soluble TLRs (sTLR) have been detected in several body fluids^11,12^. Micro RNAs (miRNAs) have been reported as antiviral regulators of viral genes^13^. MiRNAs are single-strand, non-coding, short RNAs that can regulate gene expression^14^. They play a crucial role in the pathogenesis of viral infections, particularly, coronavirus, which can alter host immune-related circulating miRNAs and causes dysregulation of the immune system^15^. Additionally, several circulating miRNAs can inhibit S protein expression and SARS-CoV-2 replication^16^. Recently, important bioinformatics-based studies have been assessed the role of miRNAs in SARS-CoV-2 infection, COVID-19 prognosis, and as potential anti-viral treatment^17,18^. Several studies have shown that altered expression of circulating miRNAs in COVID-19 patients may be a diagnostic biomarker^19^. Interestingly, six anti-viral miRNAs, namely, hsa-miR-1-3p, hsa-miR-17-5p, hsa-miR-199a-3p, hsa-miR-429, hsa-miR-15a-5p, and hsa-miR-20a-5p, were reported to play a role in respiratory viral infections, such as influenza A, and respiratory syncytial virus (RSV) and adenovirus 2^20^. MiR-20a, was also implicated in ARDS-related pathways^20^.The diagnostic ability of combined miR-20a and miR-106a serum levels to understand the pathogenesis of emerging virus infections like avian influenza was identified^21^. MiR-106a play a role in immune response as well as suppressing pro-inflammatory genes^22^. However, to date, little is known about the role of circulating miRNAs as biomarkers in COVID‐19 patients. Therefore, the previous findings prompted us to study some of these miRNAs (miR-106a and miR-20a) expression levels in serum of COVID‐19 patients compared to normal persons. Additionally the study aimed to know which cytokines were involved in cytokine storm of COVID-19 and the role of these biomarkers in disease progression.

## Results

### Demographic and clinical data of COVID-19 patients and healthy controls

The mean age of the patients and control groups was 55.9 ± 1.8 and 54.8 ± 1.2 years, respectively. Out of 50 patients 28 (56%) were males and 22 (44%) were females. Out of 30 controls 17 (56.7%) were males and 13(43.3%) were females. Out of 50 COVID-19 patients, 32 (64%) have had lymphopenia, 27 (54%) were having CT severity score (CSS) > 19 and 24 (48%) were having O2 saturation less than 90%. The clinical data of patients was summarized in supplementary (Table S1).

### Expression levels of miRNA-106a and miRNA-20a in COVID-19 patients and controls

**Figure 1:**
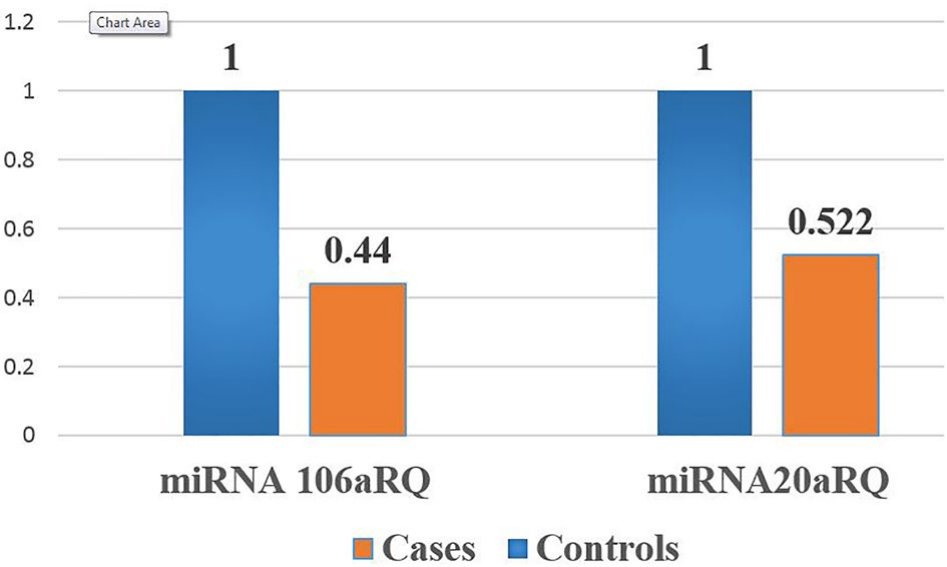
The relative expression levels of serum miRNA-106a and miRNA-20a in COVID-19 group and control group. Expression pattern of miRNA-106a and miRNA-20a in COVID-19 group compared to controls: Data were analyzed by Mann–Whitney U test, SPSS software version 22. The data was statistically significant (*p *< 0.0001**).

The results showed a highly significant decrease (*P* value = 0.0001) in the expressions of miRNA-106a and miRNA-20a in COVID-19 group compared to control group (Fig. 1).

The results showed a significant decrease in the expression levels of miRNA-20a in patients with lymphopenia compared to patients without lymphopenia (*P* value = 0.03), patients having CSS > 19 compared to patients with CSS < 19 (*P* value = 0.005) and in patients having O_2_ saturation less than 90% (*P* value = 0.02). However the expression levels of miRNA-106a showed non-significant differences (Table 1).

**Table 1:** The mean value ± SEM of serum miRNA-106a and miRNA-20a expression levels in relation to lymphopenia, chest CT severity score (CSS) and O_2_ saturation in COVID-19 patients.

Data	Present (no = 32)	Absent (no = 18)	*P* value
Lymphopenia			
miRNA-106a			
Range	0.007–2.6	0.007–1.95	0.5
Mean ± SEM	0.652 ± 0.08	0.49 ± 0.1	
miRNA-20a			
Range	0.0004–3.04	0.0013–3.18	0.03*
Mean ± SEM	0.356 ± 0.1	0.818 ± 0.2	

Data	More than 19 (no = 27)	Less than19 ( no = 23)	*P* value
Chest CT severity score (CSS)			
miRNA-106a			
Range	0.007–2.6	0.045–1.95	0.6
Mean ± SEM	0.69 ± 0.09	0.47 ± 0.09	
miRNA-20a			
Range	0.0004–1.3	0.01–3.18	0.005*
Mean ± SEM	0.25 ± 0.06	0.83 ± 0.2	

Data	Less than 90% (no=24)	More than 90% (no=26)	*P* value
O_2_ saturation			
miRNA-106a			
Mean ± SEM	0.38 ± 0.08	0.49 ± 0.1	0.4
miRNA-20a			
Mean ± SEM	0.29 ± 0.09	0.73 ± 0.1	0.02*

*Significant level at *P* value < 0.05.

### Diagnostic accuracy of serum miRNAs as potential biomarkers for COVID-19 disease

**Figure 2:**
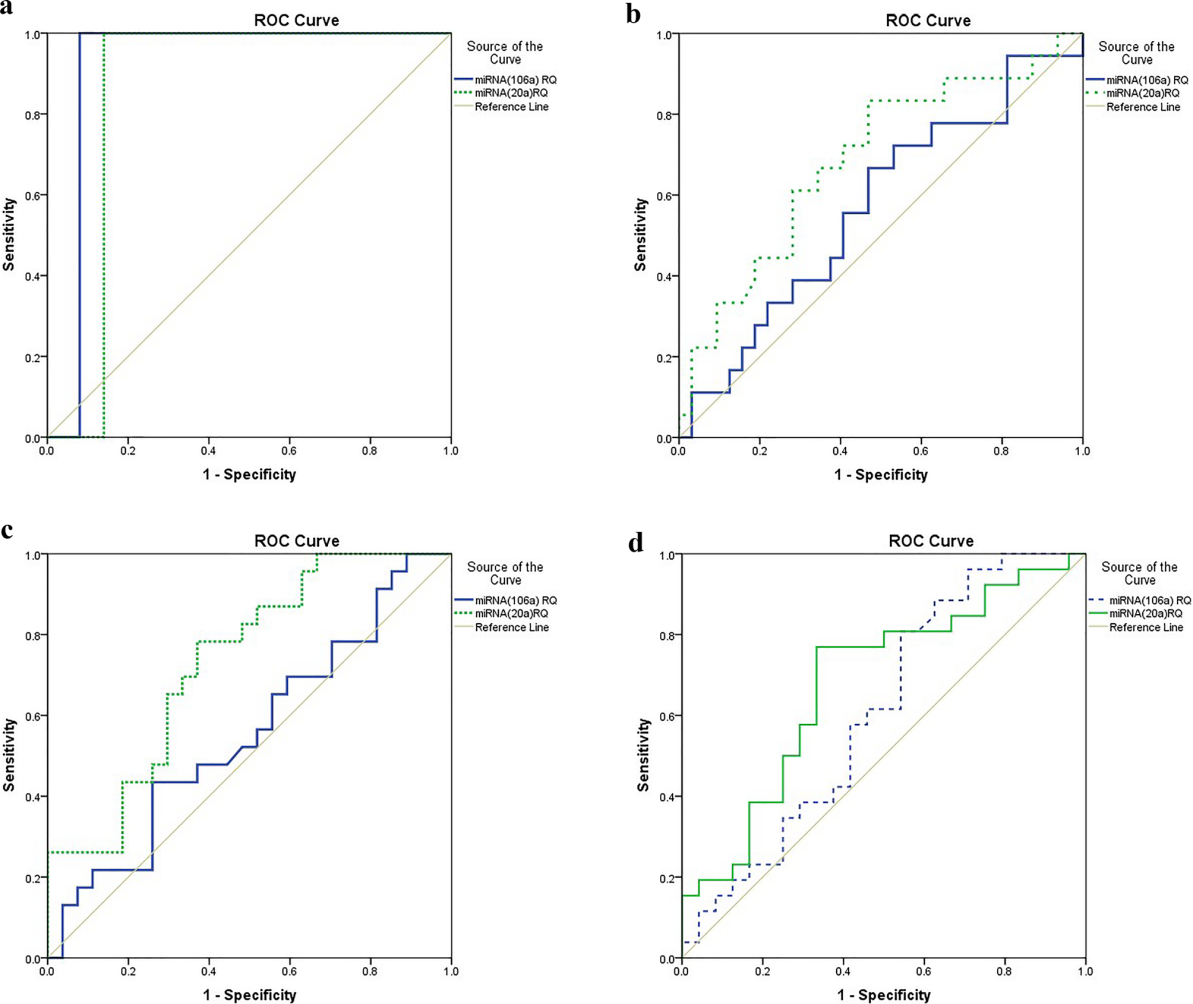
(**a**) ROC curve analysis of miRNAs of patients versus controls. Serum miRNAs as diagnostic biomarkers discriminating COVID-19 patients from healthy controls. MiRNA-106a and MiRNA-20a showed good discriminating efficiency. MiRNA-106a was downregulated in patients with AUC of (0.92 ± 0.03 *p *= 0.0001**). MiRNA-20a was downregulated in patients with AUC of (0.86 ± 0.04 *p *= 0.0001**). SPSS software version 22 was used. (**b**) ROC curve analysis for the accuracy of the association between the expression level of serum miRNAs and lymphopenia in COVID-19 patients.Serum miRNA-106a and miRNA-20a as diagnostic biomarkers to identify patients with lymphopenia compared to patients with normal lymphocytic count. MiRNA-20a showed good discriminating efficiency with AUC = (0.68±0.08, *p *=  0.03*), while miRNA-106a showed no discriminating efficiency with AUC = (0.54±0.08, *p *=  0.4). SPSS software version 22 was used. (**c**). ROC curve analysis for the accuracy of the association between the expression level of serum miRNAs and the chest CT severity score (CSS) in COVID-19 patients. Serum miRNA-106a and miRNA-20a as diagnostic biomarkers for patients with CSS >19 compared to patients with CSS >19. MiRNA-20a showed good discriminating efficiency with AUC = (0.73±0.07, *p *=  0.005*), while miRNA-106a showed no discriminating efficiency with AUC = (0.55±0.08, *p *=  0.5). SPSS software version 22 was used. (**d**) ROC curve analysis for the accuracy of the association between the expression level of serum miRNAs and O2 Saturation. Serum miRNA-106a and miRNA-20a as diagnostic biomarkers for patients with O2 Saturation > 90% compared to patients with O2 Saturation < 90%. MiRNA-20a showed good discriminating efficiency with AUC = (0.68±0.07, *p *=  0.02*), while miRNA-106a showed no discriminating efficiency with AUC = (0.61±0.08, *p *=  0.1). SPSS software version 22 was used.

Receiver operator characteristic (ROC) curve analysis was performed to assess the accuracy of the serum miRNAs in diagnosis of COVID-19 disease. ROC curve showed that miRNA-106a and miRNA-20a downregulation in COVID-19 patients compared to normal persons could be potential diagnostic markers with (AUC = 0.90 ± 0.03; *P* value = 0.0001*)) for miRNA-106a and (AUC = 0.86 ± 0.04; *P* value = 0.0001*)) for miRNA-20a (Fig. 2a). ROC curve showed also that the significant downregulation of miRNA-20a in patients having lymphopenia with (AUC = 0.68 ± 0.08; *P* value = 0.03*)) (Fig. 2b) and also in patients with CSS > 19 with (AUC = 0.73 ± 0.07; *P* value = 0.005*) (Fig. 2c) could be diagnostic. Additionally the significant downregulation of miRNA-20a in patients having O_2_ saturation less than 90% with (AUC = 0.68 ± 0.07; *p *= 0.02*) (Fig. 2d) could be a diagnostic potential biomarker.

### Inflammatory markers of COVID-19 patients and healthy controls

Serum levels of CRP, ferritin and D-dimer were measured in both groups and the results showed a highly significant increase of their mean levels in COVID-19 patients group compared to the healthy control group (*P* value = 0.0001*)) (Table 2). Measurement of serum levels of IL-10, TNF-α, INF-γ and TLR-4 showed a highly significant increase in their levels in the COVID-19 patients compared to controls (*P* value = 0.0001*) (Table 2). The results showed also a highly significant increase in the concentration levels of serum IL-10 (*P* value = 0.04*)) and serum TLR-4 (*P* value = 0.006*)) in patients having lymphopenia (Table 3). Additionally, the results showed a highly significant increase in the concentration levels of serum TLR-4 in patients having CSS > 19 (*P* value = 0.003*) (Table 4) and in patients having O_2_ saturation less than 90% (*P* value = 0.04) (Table 5).

**Table 2:** The mean value ± SEM of the concentration levels of serum biomarkers in patients and controls.

Data	Control group (no = 30)	COVID-19 group (no = 50)	*P* value
IL-10 (Pg/ml)			
Range	4.99–28	9.85–31.45	0.0001*
Mean ± SEM	13.4 ± 1.2	22.6 ± 0.7	
TNF-α (Pg/ml)			
Range	9.6–15.51	12.2–25.89	0.0001*
Mean ± SEM	11.9 ± 0.3	18.009 ± 0.5	
INF- γ (Pg/ml)			
Range	14.99–27.34	20.63–43.82	0.0001*
Mean ± SEM	20.2 ± 0.7	29.8 ± 0.9	
TLR-4 (ng/ml)			
Range	0.52–1.23	0.67–2.98	0.0001*
Mean ± SEM	0.80 ± 0.03	1.4 ± 0.1	
CRP (mg/L)			
Range	2–6	5–54	0.0001*
Mean ± SEM	3.6 ± 0.2	33.04 ± 2.7	
Serum Ferritin (ng/ ml)			
Range	95–254	144–971	0.0001*
Mean ± SEM	151.2 ± 9.8	532.9 ± 35.8	
D-dimer (μg/ml)			
Range	0.15–0.50	0.23–2.98	0.0001*
Mean ± SEM	0.30 ± 0.01	1.16 ± 0.1	

*Significant level at *P* value < 0.05.

**Table 3:** The concentration levels of serum IL-10, TNF-α, INF-γ and TLR-4 in relation to the presence of lymphopenia.

Data	Lymphopenia		*P* value
	Present (no = 32)	Absent (no = 18)	
IL-10 (pg/ml)			
Range	12.83–31.45	9.85–30.23	0.04*
Mean ± SEM	23.8 ± 0.9	20.4 ± 1.3	
TNF-α (Pg/ml)			
Range	12.7–25.3	12.1–25.8	0.7
Mean ± SEM	18.1 ± 0.7	17.8 ± 1.01	
INF- γ (Pg/ml)			
Range	21.2–40.7	20.6–43.8	0.8
Mean ± SEM	29.7 ± 1.001	30.05 ± 1.8	
TLR-4(ng/ml)			
Range	0.71–2.98	0.67–1.99	0.006*
Mean ± SEM	1.6 ± 0.1	0.98 ± 0.07	

*Significant level at *P* value < 0.05.

**Table 4:** The concentration levels of serum IL-10, TNF-α, INF-γ and TLR-4 in relation to the chest CT severity score (CSS).

Data	Chest CT severity score (CSS):		*P* value
	More than 19 (no = 27)	Less than 19 (no = 23)	
IL-10 (pg/ml)			
Range	13.32–30.23	9.85–31.4	0.8
Mean ± SEM	22.5 ± 1.04	22.7 ± 1.2	
TNF-α (Pg/ml)			
Range	14.03–25.3	12.1–25.8	0.7
Mean ± SEM	17.7 ± 0.5	18.3 ± 1.07	
INF- γ (Pg/ml)			
Range	21.2–40.7	20.6–43.8	0.6
Mean ± SEM	30.2 ± 0.9	29.3 ± 1.6	
TLR-4(ng/ml)			
Range	0.77–2.98	0.67–2.66	0.003*
Mean ± SEM	1.69 ± 0.16	1.05 ± 0.11	

*Significant level at *P* value < 0.05.

**Table 5:** The mean value ± SEM of the concentration levels of serum IL-10, TNF- α, INF- γ and TLR-4 in relation to O_2_ saturation in COVID-19 patients.

Data	O_2_ saturation less than 90% (no=24)	O_2_ saturation more than 90% (no=26)	*P* value
	Mean ± SEM	Mean ± SEM	
IL-10 (pg/ml)	21.9 ± 1.2	23.1 ± 1.02	0.4
TNF-α (Pg/ml)	18.2 ± 0.8	17.7 ± 0.8	0.7
INF- γ (Pg/ml)	31.5 ± 1.3	28.2 ± 1.2	0.06*
TLR-4 (ng/ml)	1.6 ± 0.1	1.1 ± 0.6	0.04*

*Significant level at *P* value < 0.05.

### The accuracy of the cytokines levels as potential biomarkers in COVID-19 disease

**Figure 3:**
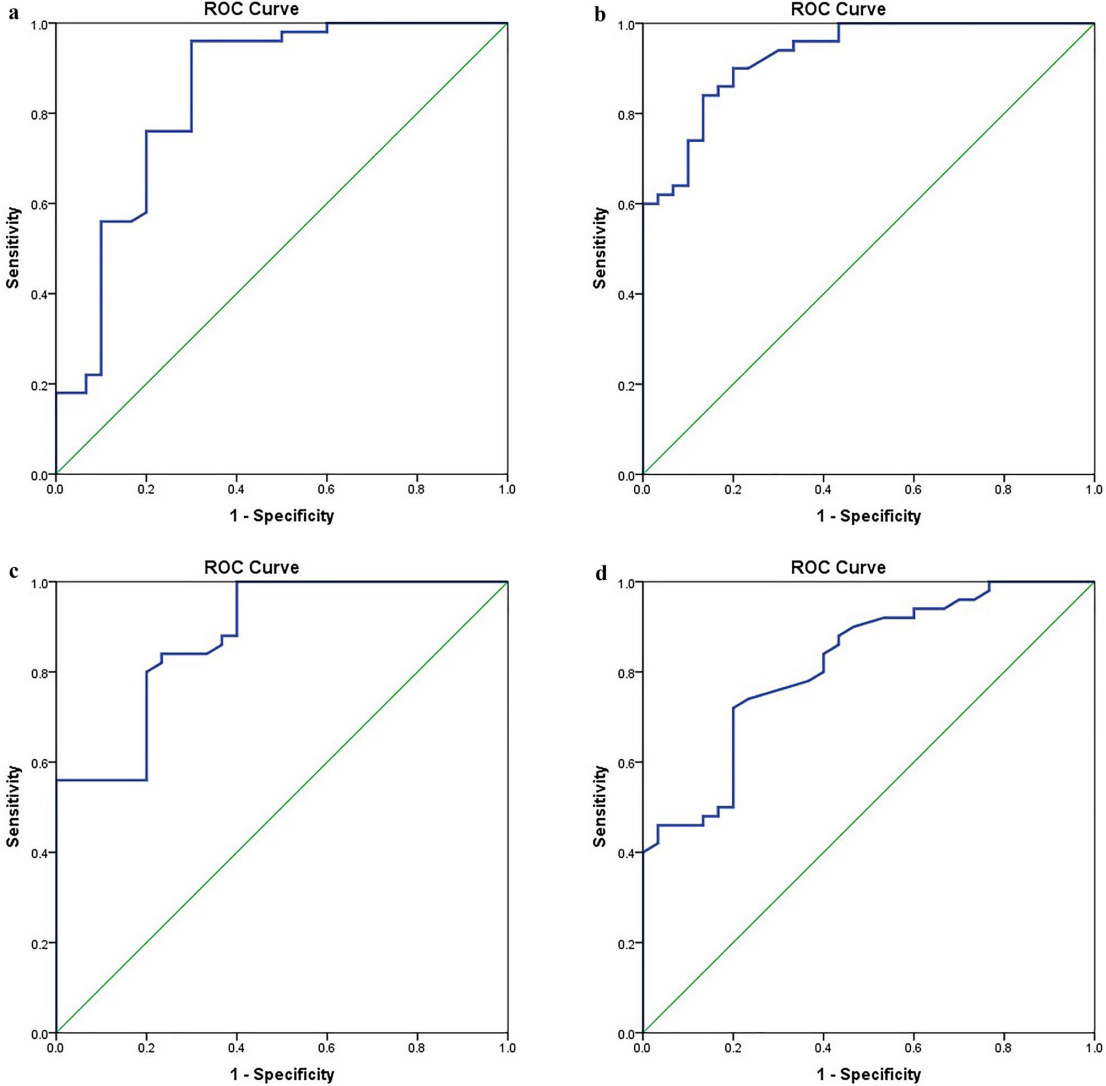
(**a**) ROC curve analysis for serum level of IL-10 in patients versus controls. IL-10 increased in patients compared to controls with good discriminating efficiency with AUC = (0.84±0.05 *p *=  0.0001*). SPSS software version 22 was used. (**b**) ROC curve analysis for serum level of TNF-α in patients versus controls. TNF-α increased in patients compared to controls with good discriminating efficiency with AUC = (0.92±0.02 *p *=  0.0001*). SPSS software version 22 was used. (**c**) ROC curve analysis for serum level of INF- γ in patients versus controls. INF- γ increased in patients compared to controls with good discriminating efficiency with AUC = (0.88±0.03 *p *=  0.0001*). SPSS software version 22 was used. (**d**) ROC curve analysis for serum level of TLR-4 in patients versus controls. TLR-4 increased in patients compared to controls with good discriminating efficiency with AUC = (0.81±0.04 *p *=  0.0001*). SPSS software version 22 was used.

**Figure 4:**
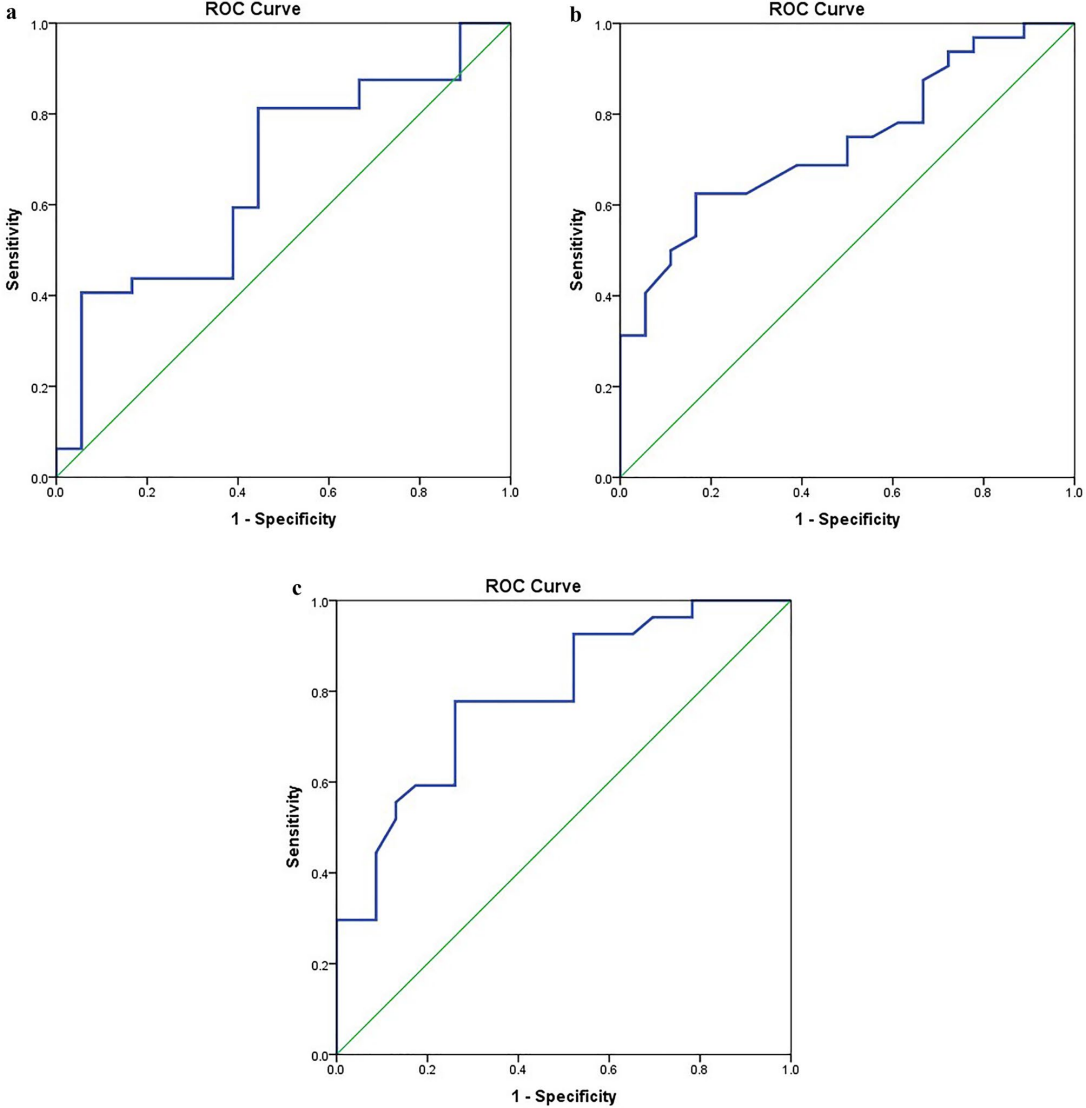
(**a**) ROC curve analysis for the accuracy of the association between serum level of IL-10 and lymphopenia in COVID-19 patients. SPSS software version 22 was used. IL-10 increased in patients with lymphopenia compared to patients with normal lymphocytic count with good discriminating efficiency with AUC = (0.66±0.08 *p *=  0.05*). SPSS software version 22 was used. (**b**) ROC curve analysis for the accuracy of the association between serum level of TLR-4 and lymphopenia in COVID-19 patients. TLR-4 increased in patients with lymphopenia compared to patients with normal lymphocytic count with good discriminating efficiency with AUC = (0.73±0.07 *p *=  0.006*). SPSS software version 22 was used. (**c**) ROC curve analysis for the accuracy of the association between serum level of TLR-4 in COVID-19 patients and chest CT severity score (CSS). Serum level of TLR-4 increased in patients with CSS >19 compared to patients with CSS >19 with good discriminating efficiency with AUC = (0.78±0.06 *p *=  0.0001*). SPSS software version 22 was used.

ROC curve analysis showed that the highly significant increase in the concentration levels of serum IL-10, TNF-α, INF- γ and TLR-4 in patients compared to normal persons could be potential accurate diagnostic markers with (AUC = 0.84 ± 0.05; *P* value = 0.0001*) for IL-10 (Fig. 3a), (AUC = 0.92 ± 0.02; *P* value = 0.0001*) for TNF-α (Fig. 3b), (AUC = 0.88 ± 0.03; *P* value = 0.0001*) for INF- γ (Fig. 3c) and (AUC = 0.88 ± 0.03; *P* value = 0.0001*) for TLR-4 (Fig. 3d). ROC curve was used also to assess the accuracy of the association between the increase of serum IL-10 and TLR-4 and the presence of lymphopenia among COVID-19 patients. The results showed that the elevation in levels of serum IL-10 and TLR-4 could be a potential diagnostic marker for lymphopenia in COVID-19 patients with (AUC = 0.66 ± 0.08; *P* value = 0.05) for IL-10 and (AUC = 0.73 ± 0.07; *P* value = 0.006) for TLR-4 (Fig. 4a and 4b). ROC curve showed also that the elevation of serum TLR-4 could be a potential diagnostic marker of high CSS among COVID-19 patients with (AUC = 0.78 ± 0.06; *P* value = 0.0001*) (Fig. 4c), but the association with hypoxia was non-significant with (AUC = 0.63 ± 0.08 *p *= 0.1).

### Simple logistic regression analysis of levels of miRNAs and cytokines in COVID-19 patients compared to controls

Simple logistic regression analysis for miRNA-106a and miRNA-20a expression levels showed that the significant decrease in the expression levels of both miRNAs in COVID-19 patients compared to controls have accurate significant predictive values. It also showed that, the significant increases in the serum IL-10, TNF-α, INF-γ and TLR-4 concentration levels in COVID-19 patients have accurate significant predictive values (Table 6).

**Table 6:** Univariate logistic regression analysis for the study biomarkers in in COVID-19 patients versus controls.

Data	Odds ratio	95% CI for odds	*P* value
miRNA 106a	0.01	0.002–0.09	0.0001*
miRNA 20a	0.26	0.10–0.66	0.005*
IL-10	1.2	1.1–1.3	0.0001*
TNF-α	2.5	1.6–3.9	0.0001*
INF-γ	1.4	1.2–1.7	0.0001*
TLR-4	82.4	5.7–1339.6	0.002*

*OR* Odds ratio, *CI* Confidence interval.

Significant level at *P* value < 0.05.

### Correlation between the expression levels of serum miRNAs and the biochemical profile of COVID-19 patients

The correlation between serum miRNA-106a and miRNA-20a expression levels with different biochemical Parameters was assessed using Pearson Correlation test. A negative correlation was detected between miRNA-20a with TLR-4 (r = − 0.30, *P* value = 0.03). The data is summarized in Table S2.

## Discussion

MiRNAs have represented as potential targets that could help to understand the mechanisms of COVID-19 infection and design novel drugs against this virus^23^. In the current study, expression levels of miR-20a and miR-106a were quantified in COVID-19 patients compared to healthy controls. MiR-20a was significantly decreased in the COVID-19 patients compared to controls. ROC curve analysis confirmed the accuracy of this result with *P* value = 0.0001*, that agreed with Giannella et al., 2022 and Li et al. 2020*,* who also reported that miR-20a is significantly downregulated in COVID-19 patients compared to the healthy controls^24,25^. However, studies on other chest diseases showed different findings; Liu et al. reported that miR‑20a was up-regulated in pediatric pneumonia and in human lung adenocarcinoma A549 cells^26^. Also Xu et al. found that miR-20a was increased in patients with non-small cell lung cancer (NSCLC) than healthy controls^27^. The expression level of miR-106a in the COVID-19 group was significantly decreased compared to the control group, that is confirmed by Roc curve (*P* value = 0.0001*). To best of our knowledge the present report is the first time to evaluate the expression level of miR-106a in the COVID-19 patients compared to healthy controls. However, these findings may be comparable with findings reported by Yang et al., 2019, as they showed that miR-106a is significantly down-regulated in the lung tissues of mice and in macrophages in induced acute lung injury^28^. Down regulation of miR-106a was also reported in T helper 17 cell (Th17) differentiations that secrete IL-17 cytokine^29^. IL-17 is important in activating neutrophils that can accumulate in the lung, and are involved in the pathogenesis of sever phenotype of COVID-19 disease^30^. Lymphopenia is associated with increased COVID-19 disease severity and associated with high mortality^31,32^. The study showed a significant decrease in serum level of miR-20a of patients with lymphopenia compared to patients with normal lymphocytic count, while the level of miR-106a showed non-significant difference. Yang et al. suggested that the severity of chest CT affection could be used to evaluate the severity of pulmonary involvement in patients with COVID- 19 infection^33^. In our study, there was a highly significant decrease in miR-20a in COVID-19 patients having CSS > 19 compared to patients with CSS < 19 and also in patients with hypoxia, while the level of miR-106a showed non-significant decrease. These findings are parellel to the findings of previous reports that identified downregulation of miR-20a in sever cases^24,25^. In the same context, Chen et al. have reported that, miR-20a could reduce inflammation by regulating TLR4 and TXNIP signaling pathways that agrees with our results, where miR-20a expression was decreased with lung affection^34^. So we could conclude that miR-20a might play a role in the pathological process of COVID-19 disease, and should be evaluated for early diagnosis of patients with more severe disease. For unclear reasons, SARS-CoV, MERS-CoV and SARS-CoV-2 induce exaggerated immune responses leading to a cytokine storm that are associated with severe lung pathology, multi-organ failure and death^32,35^. Pro-inflammatory cytokines are the main participants in the cytokine storm; IL-1β, IL-6, IL-8, IL-18, IFN-γ, TNF-α^35^. Han et al. found that the serum concentration levels of TNF-α, IFN-γ, IL-6 and IL-10 were elevated significantly in COVID-19 patients in contrast to healthy controls^36^. Also Bayraktar et al. showed that IL-10 and TNF-α levels in the COVID-19 patients were significantly higher than controls^37^. These findings are compatible with our study, where IL-10, TNF-α and INF-γ showed a highly significant increase in COVID-19 group in contrast to control group. Our results are also in agreement with Taghiloo et al. who have been reported that, the serum levels of TNF-α and INF-γ were significantly higher in COVID-19 group compared to controls, but they disagree with our findings about serum level of IL-10 as they found that the increase in level of IL-10 was statistically non-significant^38^. Several studies showed elevated levels of IL-10 predict poor outcomes in COVID-19 patients^36,39,40^. Based on its well-established role of IL-10 as an anti-inflammatory cytokine, the dramatic elevation in IL-10 could be explained as an attempt to suppress inflammation and prevent tissue injury^41^. However, the concurrent increase in IL-10, particularly with bad prognosis, suggest that IL-10 is either failing to suppress inflammation^42^ or acting in a deviated manner^43^. The binding of the spike protein to TLR4 may has a role in SARS-CoV-2 entry into human cells and initiating the cytokine storm^10^. Additionally, TLR4 signaling was identified as a key pathway of acute lung injury^44^. The current study found that*,* serum level of TLR4 was higher in COVID-19 group compared to control group (*P* value = 0.0001). Our finding agreed with Sohn et al., who reported that the expression of TLR4 and its signaling mediators were significantly upregulated in COVID-19 patients compared to healthy controls^45^. These findings strengthen the suggestion of a relation between increased TLR4 expression and its activation by the SARS-CoV-2 virus^9^. Therefore, evaluating the role played by TLR4 signaling in the lungs is important to improve the prognosis of COVID-19 disease.

MiR-20a-target several pro-inflammatory cytokine genes (e.g., *TNF, CCL2, CXCL9, IL10*) as well as cytokine and chemokine receptors (*IL1R1, IL2RA, IFNAR2*)^26^, that can explain the presence of downregulation of miR-20a and increase in cytokine levels in patients compared to control. Since miRNAs inhibit their target gene expression, the altered miRNAs in COVID-19 infection would lead to the induction of inflammation and to the inhibition of antiviral response^24^ Our findings are compatible with previous reports that demonstrated, down regulation of miR-106a increase inflammatory cytokines, including TNF-α , IL-1β and IL-6^28^. Additionally, our results agree with reports demonstrated that, increase in TLR4 is associated with downregulation of miR-106a and miR-20a^28,34^. In the current study have been studied the relation between the levels of serum IL-10, TNF-α, INF-γ and TLR-4 and the presence of lymphopenia in COVID-19 patients group. The results showed significant increases in the levels of serum IL-10 and TLR-4 in patients having lymphopenia compared to patients with normal lymphocytic count. SARS-CoV-2 might inactivate the lymphocytes or destroy lymphoid organs. Lymphopenia also may be caused by metabolic disturbances occur with severe form of COVID-19^46^. The proinflammatory cytokines are increasingly released by different immune cells and also endothelial cells to compensate the exhausted lymphocytes^47^. Zhao et al. showed that, CT involvement score can help in evaluation of the severity and extent of the COVID-19 disease^48^. A highly significant increase in the level of serum TLR-4 in COVID-19 patients with CSS > 19 compared to patients with CSS < 19 and also in patients with hypoxia was observed in the current study. A negative correlation was reported between serum miR-20a with TLR-4 (r = − 0.30, *P* value = 0.03), that agrees with Chen et al., who reported that, miR-20a could negatively regulate TLR4^34^. So further studies on the relationship between miR-20a, TLR4 and the severity of COVID-19 disease may be valuable to overcome COVID-19 pandemic. Identifying the variations of different cytokines and their relations to clinical conditions can introduce these cytokines as valuable biomarkers^49^. Early control of the cytokine storm is important to improve the disease prognosis^35^. Our current study concluded that, miR-20a, IL-10 and TLR4 are potential biomarkers for severity of COVID-19 disease and also blockade of IL-10 and TLR4 may constitute a novel therapy for COVID-19 patients.

## Materials and methods

### Study design

The current study included 50 patients, aging between (22 and 78) years old and of both sexes, with symptoms of COVID-19 pneumonia and positive reverse transcriptase-polymerase chain reaction (RT-PCR) test who admitted to the Minia university hospital. Pregnant women, Patients with immunological diseases, heart diseases, tumors, bronchial asthma or other chronic obstructive pulmonary disease [COPD] and diabetes were excluded from the study. Clinical data were collected from their medical records and an RT-PCR test was performed for all patients. All patients underwent a computed tomography (CT scan) (BrightSpeed CT scanner; GE Healthcare Systems, Milwaukee, Wisconsin, USA) according to national guideline recommendations for management of the disease within the first 24 h of hospitalization. The time gap between initial clinical assessment, CT scan and laboratory test sampling was about 24 h for all of the study patients. CT severity score (CSS) was calculated by a senior radiologist as presented previously^50^. Thirty healthy volunteers, matched in age and sex with patients were also included in the study as a control group. The study participants had no history of COVID-19 vaccination. The study was conducted from December 2020 to June 2021 according to the guidelines for the use of human subjects’ materials of the “Declaration of Helsinki.” The study protocol was accepted by the Research Ethics Committee (REC) of Minia university hospital, Egypt (693:11/220). Written informed consents were obtained from all participants.

### Samples collection and biochemical assessment

Under complete aseptic conditions, blood samples (6 mL) were collected from the study population. Serum was obtained by centrifugation and was immediately frozen at − 80 °C.

D-dimer was determined using a Sysmex 5100 analyzer (Siemens Healthcare Diagnostics, Marburg, Germany), while CRP and ferritin were determined using a Cobas analyzer (Roche Diagnostics GmbH, Mannheim, Germany). Complete blood count (CBC) was carried out by automated cell counter (Sysmex KX-21N, TAO Medical Incorporation, Japan). Serum level of IL-10 (AssayGenie Co., Ltd. Ireland, Catalogue Code: HUFI00156), interferon-gamma (IFN-γ) (Wuhan Fine Biotech Co., Ltd. China, Catalogue Number: EH0164), tumor necrosis factor-alpha (TNF-α) (Hcusabio Biotech Co., Ltd. China, Catalog Number: CSB-E04740h) and toll like receptor 4 (TLR-4) (Hcusabio Biotech Co., Ltd. China, Catalog Number: CSB-E12954h) were examined by ELIZA according to the manufacturer's instructions. COVID-19 viral RNA was quantified by reverse transcriptase polymerase chain reaction (RT-PCR) (Qiagen, Hilden, Germany) following extraction of RNA using QIAmp Viral RNA extraction Kit (Qiagen, Santa Clarita, CA).

### Serum miRNAs extraction and quantitation of miRNAs

Total RNA with preserved miRNAs was extracted from a volume of 200 μL serum of each participant using the miRNeasy extraction kit (Qiagen, Valencia, USA). The isolated RNA was investigated using a NanoDrop-2000 spectrophotometer (Thermo Fisher Scientific, New York, USA). Reverse transcription was done on 12 µL of total RNA in a final volume of 20 μL (incubated for 60 min at 37 °C, 5 min at 95 °C and then maintained at 4 °C) using the miRNeasy serum/plasma reverse transcription Kit (Qiagen, Valencia, CA, USA). Real-time PCR quantification assays for miR-106a and miR-20a were performed using miScript sybr green master mix reagents (Qiagen, Valencia, USA). MiR-U6 gene was utilized as the internal control. All primers were supplied by Qiagen. (Qiagen, Valencia, USA). All experiments were conducted in duplicate according to the manufacturer's instructions. Applied Biosyst 7500 fast, Techne (Cambridge) LTD., UK Real-Time PCR System was used for the study experiments. The levels of miRNAs were reported as the ΔCt value that was calculated by subtracting the CT values of miR-U6 from the CT values of the target miRNAs. The equation used to calculate the fold change (relative quantity “RQ”) of miRNA levels using healthy controls as calibrator is 2^−ΔΔCt.^ ΔΔCt = ΔCT of patient – mean of ΔCT of control.

### Statistical analysis

The statistical analysis of the study was performed using statistical package for social science (SPSS) software version 22 (SPSS Inc, Chicago). The quantitative data was analyzed by the Student's t test. Chi square (X2) test was used for categorical data. Mann–Whitney was used for comparison between the different groups. Logistic regression analysis was done to identify predictor biomarkers to avoid the effects of co-linearity. *P* values < 0.05 were considered statistically significant. The receiver operating characteristic curve (ROC) analysis was used to assess the accuracy of the study results. The area under the curve ROC (AUC) identified optimal sensitivity and specificity of the study assays. Pearson correlation test was used to show the correlation between different variables.

### Research involving human participants and/or animals

The research was carried out in accordance with the 1975 Helsinki Declaration. The study protocol was accepted by the Research Ethics Committee (REC) of Minia university hospital, Egypt (693:11/220). No animals were involved in the study.

## Supplementary Information

Supplementary Information.

## Data Availability

All data generated or analyzed during this study are included in this published article [and its supplementary information files].

## Abbreviations

AUC: Area under curve
COVID-19: Coronavirus disease 2019
CT scan: Computed tomography
IL-10: Interleukin 10
miRNAs: MicroRNAs
SARS-CoV-2: Severe acute respiratory syndrome coronavirus 2
ROC curve: Receiver operating curve
TLR4: Toll like receptor 4
TNF-α: Tumor necrosis factor-α
