# Supplementary material for: MicroRNAs and cytokines as potential predictive biomarkers for COVID-19 disease progression
Source: Sci Rep. 2023 Mar 2;13:3531. doi: 10.1038/s41598-023-30474-6 (PMC9979137; doi:10.1038/s41598-023-30474-6)
Supplement: Supplementary file 1 — Supplementary Information. [file 41598_2023_30474_MOESM1_ESM.docx]

**Table (1s): Clinical data of the studied patients (total=50)**

| **P value** | **Absent**  **No (%)** | **Present**  **No (%)** | **Data** |
| --- | --- | --- | --- |
| 0.0001* | 10 (10%) | 40 (90%) | **Fever** |
| 0.0001* | 14 (28%) | 36 (72%) | **Cough** |
| 0.2 | 22 (44%) | 28 (56%) | **Wheezy chest** |
| 0.002* | 20 (40%) | 30 (60%) | **Dyspnea** |
| 0.005* | 32 (64%) | 18 (36%) | **Diabetes mellitus** |
| 0.005* | 32 (64%) | 18 (36%) | **Hypertension** |
| 0.0003* | 34 (68%) | 16 (32%) | **GIT Symptoms** |
| 0.005* | 18 (36%) | 32 (64%) | **Lymphopenia** |
| 0.4 | 23 (46%) | 27 (54%) | **Chest affection** |
| 0.0001* | 36 (72%) | 14 (28%) | **Congested throat** |
| 0.0001* | 36 (72%) | 14 (28%) | **Dry tongue** |
| 0.6 | 24 (48%) | 26 (52%) | **O2 saturation more than 90%** |
| 0.6 | 26 (52%) | 24 (48%) | **O2 saturation less than 90%** |

**Table (2S): Correlation between the expression levels of serum miRNA-106a and miRNA-20a and the biochemical profile of COVID-19 patients:**

| **miRNA20aRQ** | | **miRNA106aRQ** | | **Data** |
| --- | --- | --- | --- | --- |
| **P value** | **r** | **P value** | **r** |  |
| 0.1 | -0.19 | 0.5 | 0.07 | **CRP (mg/L)** |
| 0.7 | 0.05 | 0.1 | 0.22 | **Ferritin(ng/ml)** |
| 0.07 | -0.25 | 0.3 | -0.14 | **D-dimer(g/ml)** |
| 0.4 | 0.10 | 0.4 | 0.11 | **IL-10(Pg/ml)** |
| 0.2 | -0.15 | 0.5 | 0.08 | **TNF(Pg/ml)** |
| 0.1 | -0.20 | 0.4 | 0.11 | **INF(Pg/ml)** |
| **0.03*** | **-0.30** | 0.07 | -0.22 | **TLR-4(ng/ml)** |
